# Coexistence of diffuse panbronchiolitis and sarcoidosis revealed during splenectomy: a case report

**DOI:** 10.1186/s12890-020-1117-y

**Published:** 2020-03-30

**Authors:** Tomohiro Akaba, Kiyoshi Takeyama, Mitsuko Kondo, Fumi Kobayashi, Asako Okabayashi, Tatsuo Sawada, Etsuko Tagaya

**Affiliations:** 1 Department of Respiratory Medicine, Tokyo Women’s Medical University School of Medicine, 8-1 Kawada-cho, Shinjuku-ku, Tokyo, 162-8666 Japan; 2 Division of Pathological Neuroscience, Department of Pathology, Tokyo Women’s Medical University School of Medicine, Tokyo, Japan

**Keywords:** Sarcoidosis, Diffuse panbronchiolitis, Splenectomy, CD4/CD8 ratio

## Abstract

**Background:**

Sarcoidosis is a systemic granulomatous disease caused by CD4+ cell-dominant inflammation. Meanwhile, diffuse panbronchiolitis is a chronic inflammatory respiratory disease predominantly caused by CD8+ lymphocytes and neutrophils. Herein, we report a rare case of sarcoidosis in which the clinical presentation had become evident as diffuse panbronchiolitis after splenectomy for sarcoidosis.

**Case presentation:**

A 23-year-old Japanese woman was referred to our hospital due to splenomegaly of unknown etiology. Upon admission, chest computed tomography scan revealed centrilobular and randomly distributed small nodules in both lungs. Bronchoalveolar lavage revealed a high proportion of lymphocytes and a decreased CD4/CD8 ratio. However, the biopsy specimens obtained from both the liver and lungs revealed noncaseating epithelioid granulomas, which confirmed the diagnosis of sarcoidosis. The patient underwent splenectomy due to progressive cytopenia and high risk of splenic rupture. After the surgery, the condition of the patient was consistently good for 3 months. Then, she gradually developed productive cough and dyspnea. Both sinus and chest computed tomography scan revealed chronic paranasal sinusitis and deterioration of centrilobular nodules in both lung fields, respectively. The second bronchoalveolar lavage revealed a high proportion of neutrophils, and the bronchoalveolar lavage fluid tested positive for *Hemophilus influenzae*. The titer of cold agglutinin was elevated, thereby confirming the diagnosis of diffuse panbronchiolitis. On the basis of the clinical and radiological findings, the condition of the patient improved with low-dose macrolide therapy for 3 months.

**Conclusions:**

The coexistence of sarcoidosis and diffuse panbronchiolitis has not been previously reported, and the hidden profiles of diffuse panbronchiolitis may have been revealed by splenectomy.

## Background

Sarcoidosis is a systemic granulomatous disease of unknown etiology. The lungs and intrathoracic lymph nodes are the commonly affected regions, and the liver and spleen are the most commonly affected extrapulmonary organs [1, 2]. A high proportion of lymphocytes and an elevated CD4+ to CD8+ T-lymphocyte ratio (CD4/CD8 ratio) in the bronchoalveolar lavage (BAL) fluid (BALF) indicate CD4+ cell-dominant inflammation in sarcoidosis. Radiological findings of the lungs revealed various patterns, such as bilateral hilar lymphadenopathy, presence of perilymphatic or centrilobular small nodules, and nodular thickening along the lymphatic vessels in the bronchovascular bundle [3]. Therefore, the differential diagnosis for sarcoidosis is broad and distinguishing the condition from other respiratory diseases is sometimes challenging.

Meanwhile, diffuse panbronchiolitis (DPB) is a chronic inflammatory respiratory disease predominantly affecting East Asians [4]. The typical pathological features are observed in the respiratory bronchioles, which include peribronchial infiltration of foamy histiocytes, neutrophils, and CD8+ lymphocytes. Chest computed tomography (CT) scan revealed centrilobular nodules and bronchial wall thickening, indicating chronic neutrophilic inflammation caused by *Hemophilus influenzae* or *Pseudomonas aeruginosa*.

The mechanisms of these two respiratory diseases are different, and the coexistence of sarcoidosis and DPB has not been observed in previous studies. Herein, we report a case of sarcoidosis in which the clinical presentation had become evident as DPB after splenectomy for sarcoidosis.

## Case presentation

**Fig. 1:**
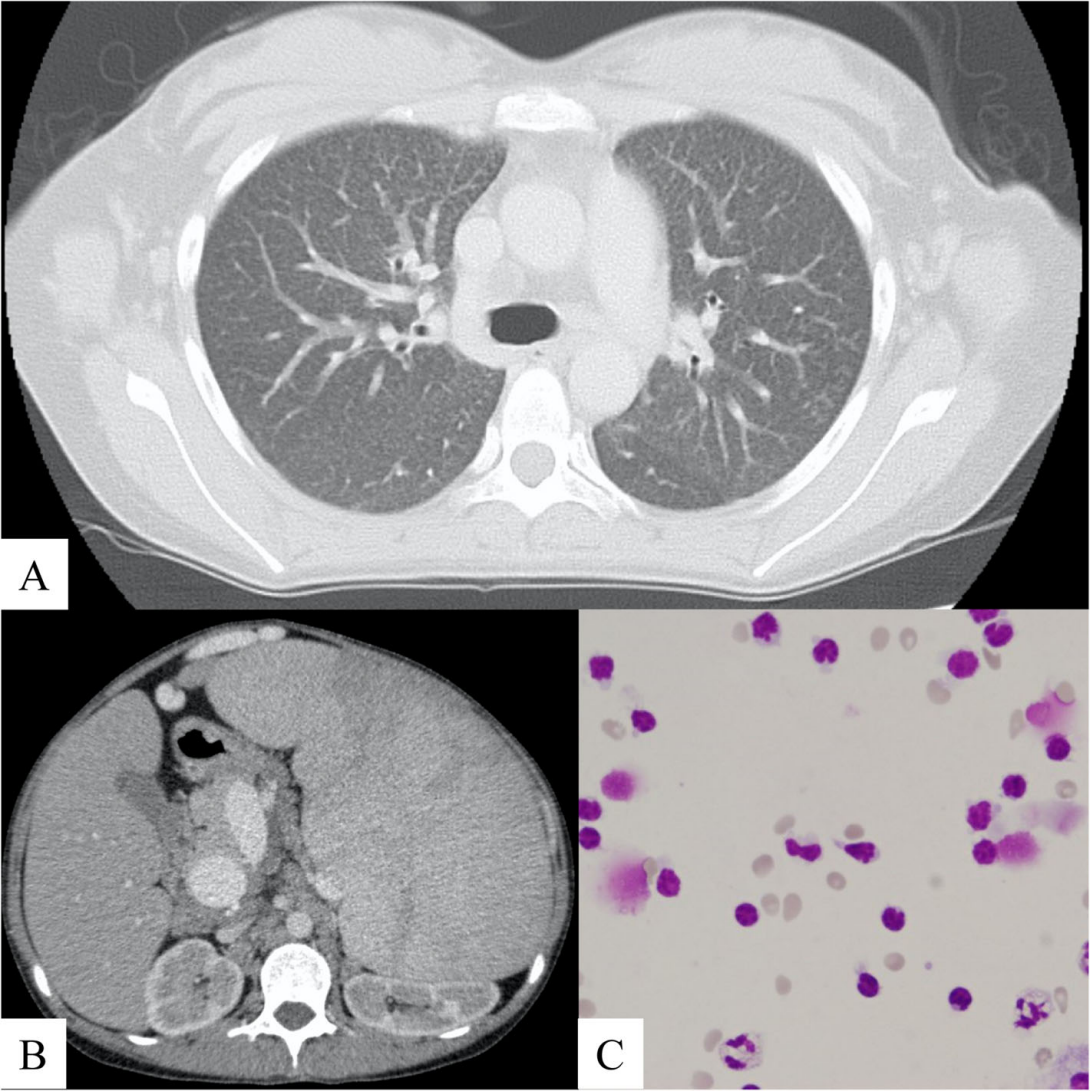
**a** Chest computed tomography (CT) scan revealed the presence of centrilobular nodules in the lung parenchyma. **b** Abdominal CT scan revealed the remarkable enlargement of the spleen. **c** The first analysis of the bronchoalveolar lavage fluid revealed a high proportion of small lymphocytes

**Fig. 2:**
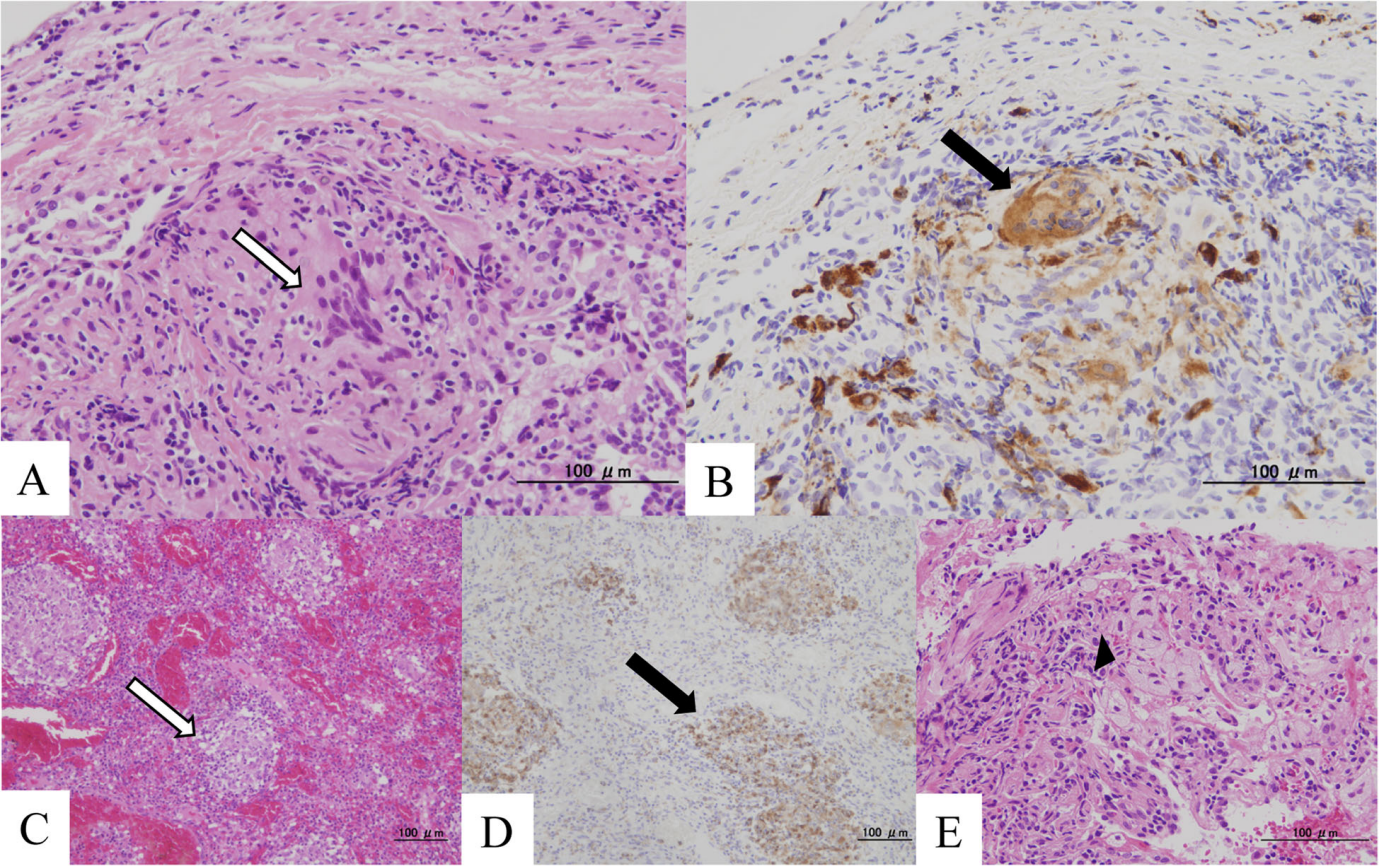
**a** The biopsy specimen from the lung showed noncaseating granuloma with accumulation of epithelioid cells (white arrow). **b** Immunohistological staining of the biopsy specimen revealed the presence of CD68-positive cells, which represented epithelioid cells (black arrow). **c** The resected spleen had diffusely scattered noncaseating epithelioid granulomas (white arrow). **d** CD68-positive cells were observed in the scattered granuloma region (black arrow). **e** Foamy cells infiltrating the interstitial wall were observed around the respiratory bronchiole (black arrowhead)

A 23-year-old Japanese woman was referred to our hospital due to splenomegaly of unknown etiology and cytopenia. Before referral, she underwent intensive examinations, including bone marrow aspiration and spleen biopsy, at other medical care centers for the detection of hematologic, endocrine, and hereditary diseases in a differential diagnosis. However, a definitive diagnosis was not made. Upon admission, the patient presented with dyspnea upon exertion and abdominal distention. Chest CT scan revealed the presence of both centrilobular and randomly distributed small nodules in both lungs (Fig. 1a). In addition, abdominal CT scan revealed splenomegaly (Fig. 1b). Lung function test upon admission revealed restrictive ventilatory impairment with reduced pulmonary diffusion capacity. Liver biopsy was performed, and the biopsy specimen revealed a noncaseating granuloma, indicating sarcoidosis. Then, the patient underwent transbronchial lung biopsy and BAL to evaluate for lung involvement. Although an increased number of small lymphocytes in the BALF, which is a typical finding, was observed, the CD4/CD8 ratio decreased to 0.12 (Fig. 1c and Table 1). Moreover, the biopsy specimen from the lung showed noncaseating epithelioid granuloma positively stained with CD68 in the lung interstitium (Fig. 2a, b). The tuberculin reaction test had a negative finding. On the basis of these results and the elevated level of angiotensin-converting enzyme (ACE) (45.1 U/L), lysozyme (36.4 μg/mL), and serum soluble interleukin-2 receptor (5290 U/mL), the diagnosis of sarcoidosis was confirmed (Table 2). Because of progressive cytopenia and high risk of splenic rupture, splenectomy was chosen as the initial therapy. The pathological findings of the resected spleen revealed noncaseating granuloma, which was consistent with the splenic lesion in sarcoidosis (Figs. 2c, d). Along with the decrease in the level of serum ACE (24.1 U/L) and lysozyme (22.4 μg/mL), dyspnea and abdominal distention improved after splenectomy (Table 3).

**Table 1:** Cell count in the first bronchoalveolar lavage fluid

BALF findings		Criterion value
total cell (/mL)	2.6 × 10*5	0.5 × 10*5 ~ 2.0 × 10*5
macrophage (%)	22.7	80 ~ 94
lymphocyte (%)	69.9	5.0 ~ 18
neutrophil (%)	5.8	0 ~ 2.0
eosinophil (%)	1.3	0 ~ 1.0
basophil (%)	0	0
mast cell (%)	0.3	0
CD4/CD8 ratio	0.12	1.0 ~ 2.0

**Table 2:** Blood test results upon admission

Laboratory findings		Criterion value
WBC (/μL)	1760	4000 ~ 8600
Neu (%)	61.3	38 ~ 70
Lymp (%)	23.9	27 ~ 45
Mono (%)	11.9	0 ~ 7
Eosino (%)	2.3	0 ~ 2
RBC (/μL)	4.22 × 10*6	3.80 × 10*6 ~ 4.80 × 10*6
Hb (g/dL)	11.1	12.0 ~ 16.0
Hct (%)	35.9	35.0 ~ 43.0
Plt (/μL)	6.5 × 10*4	15.0 × 10*4 ~ 35.0 × 10*4
TP (g/dL)	7.5	6.5 ~ 8.2
Alb (g/dL)	4.1	3.8 ~ 5.1
T-Bil (mg/dL)	0.9	0.2 ~ 1.2
AST (U/L)	45	13 ~ 33
ALT (U/L)	25	6 ~ 31
LDH (U/L)	188	119 ~ 229
ALP (U/L)	557	115 ~ 359
γ-GTP (U/L)	64	6 ~ 46
Cr (mg/dL)	0.88	0.48 ~ 0.79
BUN (mg/dL)	8.0	8.0 ~ 20.0
CRP (mg/dL)	0.73	0 ~ 0.30
ACE (U/L)	45.1	7.0 ~ 25.0
Lysozyme (μg/mL)	36.4	3.4 ~ 8.6
KL-6 (U/mL)	1134	< 500
sIL-2R (U/mL)	5290	120 ~ 500
T-SPOT	negative	negative

**Table 3:** Blood test result after splenectomy

Laboratory findings		Criterion value
ACE (U/L)	24.1	7.0 ~ 25.0
lysozyme (μg/mL)	22.4	3.4 ~ 8.6
sIL-2R (U/mL)	4114	120 ~ 500
HTLV-1	negative	negative
HIV	negative	negative
cold agglutinin test (titer)	256	4 ~ 64

**Fig. 3:**
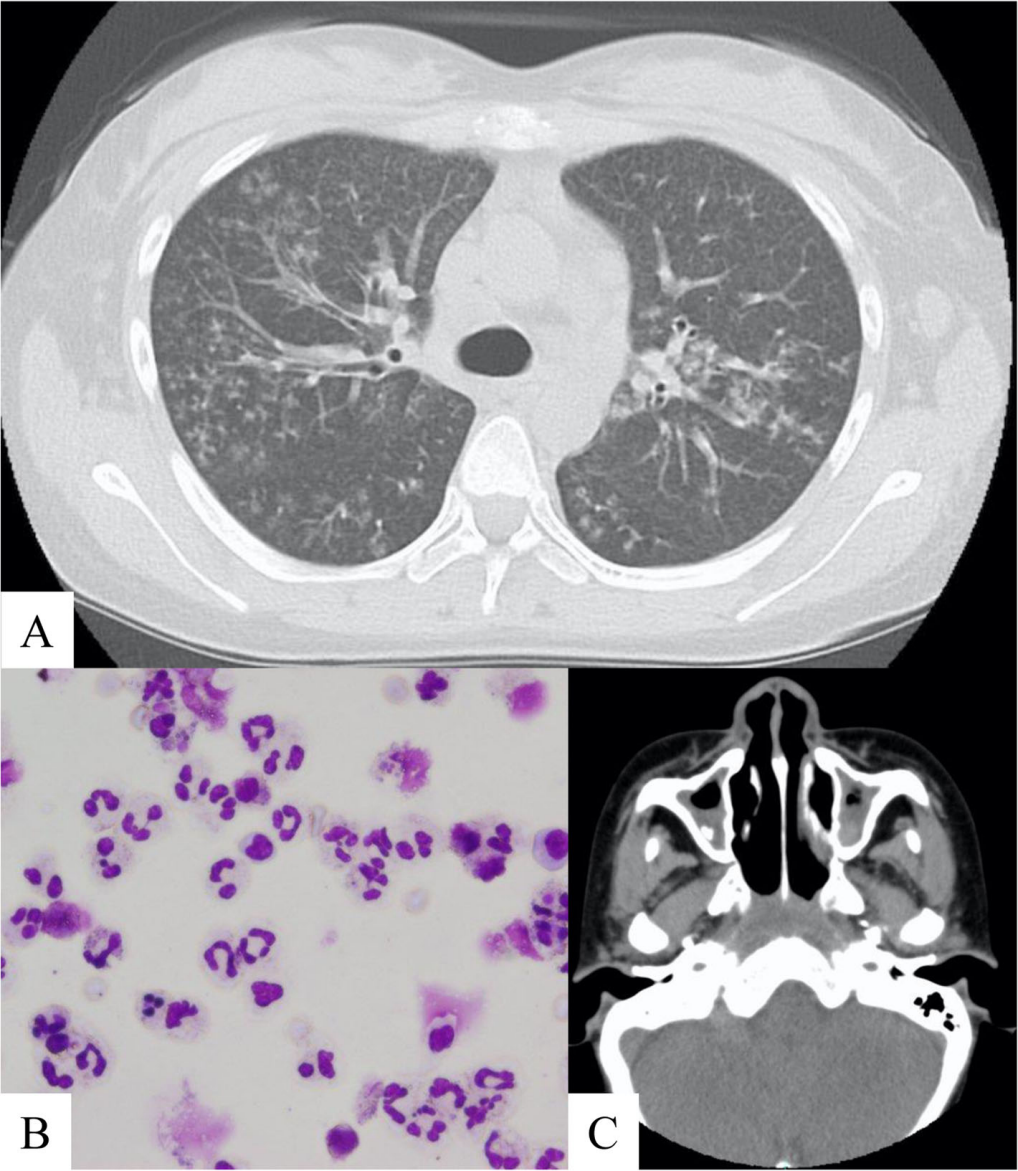
**a** Chest computed tomography (CT) scan revealed bronchial wall thickening and presence of centrilobular nodules 3 months after splenectomy. **b** The second analysis of the bronchoalveolar lavage fluid revealed a remarkable increase in neutrophil count. **c** Paranasal sinus CT scan revealed thickening of the maxillary sinus wall

**Fig. 4:**
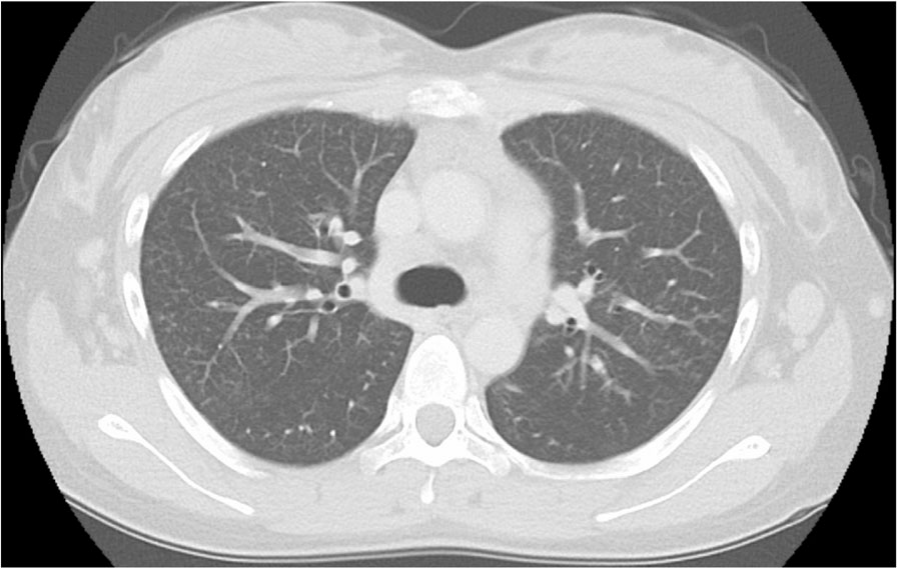
The pattern of centrilobular nodules slightly improved after the initiation of macrolide therapy

The patient complained of gradually exacerbating dyspnea and increase in purulent sputum 3 months after splenectomy. Chest CT scan revealed the presence of centrilobular nodules with bronchial wall thickening in both lung fields (Fig. 3a). The second BAL revealed a remarkable increase in neutrophil count (Fig. 3b, Table 4). The bacterial culture from the BALF tested positive for *Hemophilus influenzae.* In addition, the cold agglutinin test had positive results, and paranasal sinus CT scan revealed inflammation of the maxillary sinus (Fig. 3c). Because these data showed the characteristics of DPB, we re-evaluated the lung biopsy specimen obtained during the first bronchoscopy. The foamy cells infiltrating the interstitial wall surrounding a respiratory bronchiole supported the diagnosis of DPB (Fig. 2e). Because DPB primarily caused the patient’s current symptoms and radiological deterioration, 600 mg of erythromycin was administered daily as the initial treatment. After administrating low-dose macrolide therapy for 3 months, the patient’s condition improved based on the clinical and radiological findings (Fig. 4).

**Table 4:** Cell count in the second bronchoalveolar lavage fluid

BALF findings		Criterion value
total cell (/mL)	2.3 × 10*5	0.5 × 10*5 ~ 2.0 × 10*5
macrophage (%)	0.7	80 ~ 94
lymphocyte (%)	5.3	5.0 ~ 18
neutrophil (%)	94	0 ~ 2.0
eosinophil (%)	0	0 ~ 1.0
basophil (%)	0	0
mast cell (%)	0	0
CD4/CD8 ratio	0.09	1.0 ~ 2.0

## Discussion and conclusions

Herein, we report the coexistence of sarcoidosis and DPB, which became evident after splenectomy. The diagnosis of sarcoidosis was confirmed on the basis of the presence of noncaseating granulomas in the biopsy specimens obtained from several organs, including the liver, spleen, and lungs. Increased lymphocyte count in the BALF is commonly observed in patients with sarcoidosis; the CD4+ lymphocytes infiltrate into the alveoli, causing the high CD4/CD8 ratio in the BALF [1, 2]. However, the CD4/CD8 ratio in the BALF in the present case was low (0.12), which was not consistent with sarcoidosis. A CD4/CD8 ratio < 1 is observed in 12% of patients with sarcoidosis confirmed via biopsy [5]. However, the cause of CD8+ lymphocyte predominance in sarcoidosis is not completely understood. In the present case, DPB might have affected the CD4/CD8 ratio in the BALF. Because neutrophils and CD8+ lymphocytes play a role in the development of DPB, the migration of CD8+ lymphocytes into the alveoli might have resulted in a low CD4/CD8 ratio in the BALF [6].

Splenectomy might have triggered the exacerbation of DPB. The levels of serum ACE and lysozyme, which reflect the disease activity of sarcoidosis, decreased after splenectomy. By contrast, the presence of centrilobular nodules, which is one of the radiological features of DPB, was found on chest CT scan in the current case. Furthermore, the second BAL revealed both increased proportion of neutrophils and a positive bacterial culture for *Hemophilus influenzae*. Since splenectomy is a risk factor for infectious diseases caused by *Hemophilus influenza*, it may explain the exacerbation in DPB.

In the present case, the histological features of DPB were also confirmed; biopsy specimens obtained during the first bronchoscopy showed accumulation of foamy cells around the respiratory bronchioles where the distinctive feature is observed in patients with DPB [7]. The deterioration of bronchiolitis was successfully treated with a low-dose macrolide therapy. Similarly, a sarcoidosis case that diagnosed during the clinical course of idiopathic bronchiolitis mimicking DPB was improved by erythromycin [8].

In conclusion, the present case highlighted the complex presentation of the coexistence of sarcoidosis and DPB. If a patient with sarcoidosis presents with an atypical disease presentation, the coexistence of other diseases should be considered.

## Data Availability

All data and materials of this article are included in the manuscript and are available to the readers.

## Abbreviations

CD4/CD8 ratio: CD4+ to CD8+ T-lymphocytes
BALF: Bronchoalveolar lavage fluid
DPB: Diffuse panbronchiolitis
CT: Computed tomography
ACE: Angiotensin-converting enzyme
